# Assessment of pharmacokinetic changes of meropenem during therapy in septic critically ill patients

**DOI:** 10.1186/2050-6511-15-21

**Published:** 2014-04-14

**Authors:** João Goncalves-Pereira, Nuno Elvas Silva, André Mateus, Catarina Pinho, Pedro Povoa

**Affiliations:** 1 Polyvalent Intensive Care Unit, São Francisco Xavier Hospital, CHLO, Lisbon, Portugal, Estrada do Forte do Alto do Duque, Lisboa 1449-005, Portugal; 2 CEDOC, Faculty of Medical Sciences, New University of Lisbon, Lisbon, Portugal, Campo dos Mártires da Pátria, 130, Lisboa 1169-056, Portugal; 3 Faculty of Pharmacy, University of Lisbon, Lisbon, Portugal, Av. Prof. Gama Pinto, Lisbon 1649-003, Portugal

**Keywords:** Meropenem, β-lactam antibiotics, Pharmacokinetics, Intensive care unit

## Abstract

**Background:**

Meropenem is a carbapenem antibiotic commonly used in critically ill patients to treat severe infections. The available pharmacokinetic (PK) data has been mostly obtained from healthy volunteers as well as from clinical studies addressing selected populations, often excluding the elderly and also patients with renal failure. Our aim was to study PK of meropenem in a broader population of septic critically ill patients.

**Methods:**

We characterized the PK of meropenem in 15 critically ill patients during the first 36 hrs of therapy. Aditionally, whenever possible, we collected a second set of late plasma samples after 5 days of therapy to evaluate PK intra-patient variability and its correlation with clinical course.

Patients received meropenem (1 g every 8 hrs IV). Drug plasma profiles were determined by high-performance liquid chromatography. The PK of meropenem was characterized and compared with clinical parameters.

**Results:**

Fifteen septic critically ill patients (8 male, median age 73 yrs) were included. The geometric mean of the volume of distribution at the steady state (*V*_ss_)/weight was 0.20 (0.15-0.27) L/kg. No correlation of *V*_ss_/weight with severity or comorbidity scores was found. However the Sequential Organ Failure Assessment score correlated with the *V*_ss_/weight of the peripheral compartment (r^2^ = 0.55, p = 0.021). The median meropenem clearance (Cl) was 73.3 (45–120) mL/min correlated with the creatinine (Cr) Cl (r^2^ = 0.35, p = 0.033).

After 5 days (N = 7) although *V*_ss_ remained stable, a decrease in the proportion of the peripheral compartment (*V*_ss2_) was found, from 61.3 (42.5-88.5)% to 51.7 (36.6-73.1)%. No drug accumulation was noted.

**Conclusions:**

In this cohort of septic, unselected, critically ill patients, large meropenem PK heterogeneity was noted, although neither underdosing nor accumulation was found. However, Cr Cl correlated to meropenem Cl and the *V*_ss2_ decreased with patient’s improvement.

## Background

Meropenem is a carbapenem antibiotic with a broad antibacterial spectrum, commonly used in critically ill patients to treat severe infections. Its dose and schedule are based on pharmacokinetic (PK) data mostly obtained from healthy volunteers, as well as from clinical studies [1]. However in critically ill patients, seldom evaluated, this drug often presents different PK behaviour, and conventional dosing may fail to provide adequate antibiotic concentrations [2-4] due to both fluid shifts and therapeutic interventions. Moreover elderly patients and patients with renal failure are commonly excluded from PK studies and, therefore, it may be even more difficult to generalize the results.

Some populations, especially those with augmented renal clearance seem to be at special risk of sub-therapeutic drug concentration. Therapeutic drug monitoring has been proposed to minimize dosage inadequacy, reducing the occurrence of sub-therapeutic concentrations or drug accumulation [5]. Our purpose was to characterize the concentration time profile of meropenem in a broad population of critically ill infected patients in the early stages of infection treatment, to determine if the recommended dose resulted in adequate plasma concentrations, according to the minimal inhibitory concentration (MIC) of susceptible bacteria. We also intended to characterize PK late profile, after at least 5 days of therapy, in particular volume of distribution at steady state (*V*_ss_) and clearance (Cl), to unveil changes on drug PK behaviour during patient clinical course, and consequently the need for a dosage adjustment during therapy [6].

## Methods

The study was approved by the Ethics Committee of the Centro Hospitalar de Lisboa Ocidental. All patients or their legal representatives provided written informed consent.

Infected critically ill patients requiring intravenous meropenem (by decision of the attending physician), admitted to the intensive care unit (ICU) between May of 2009 and May of 2010, were recruited, irrespectively of comorbidities or of renal function. Only patients receiving renal replacement therapy were excluded.

All patients received 1000 mg of meropenem every 8 hrs by an intravenous central line infusion during 30 minutes. The exact duration of the infusion was registered for accurate PK calculations. The line was flushed after meropenem infusion to ensure administration of the entire vial of the drug.

Collected data (Table 1) included Simplified Acute Physiology Score (SAPS) II score [7], Sequential Organ Failure Assessment (SOFA) [8] score on the day of sample collection, Charlson comorbidity score [9], measured creatinine (Cr) Cl (in 4 hrs urine samples).

**Table 1:** Demographic and clinical data

**Patient**	**Gender**	**Age (years)**	**Weight (kg)**	**MV**	**Cr Cl (mL/min)**	**Infection focus**	**Vasop**	**Surgery**	**Charlson**	**SAPS II**	**SOFA**
1	M	73	77	No	76.7	Lung	Yes	No	4	26	4
2	F	58	55	Yes	25.0	Lung	No	No	3	44	5
3	F	77	65	No	66.7	Intra-abdominal	No	Yes	9	48	4
4	M	79	78	No	23.3	Intra-abdominal	No	Yes	6	38	2
5	F	78	85	Yes	81.7	Bacteremia	No	No	9	47	4
6	M	73	78	Yes	65.0	Unknown	Yes	Yes	5	43	8
7	F	76	80	Yes	43.3	Intra-abdominal	Yes	Yes	3	72	8
8	M	53	60	No	15.0	Skin/Soft tissue	Yes	No	6	34	6
9	F	71	90	Yes	NA	Intra-abdominal	No	Yes	6	37	3
10	M	41	80	Yes	116.7	Intra-abdominal	No	No	1	35	5
11	M	51	70	Yes	41.7	Lung	Yes	No	4	50	9
12	F	90	75	Yes	NA	Central nervous system	No	Yes	6	58	6
13	M	34	63	Yes	95.0	Lung	No	No	0	32	2
14	M	67	80	Yes	226.7	Lung	No	No	3	47	4
15	F	76	100	No	51.7	Intra-abdominal	No	Yes	3	47	2
**Median [IQR]**		73 [21]	78 [12.5]		65.0 (40)				4 [3]	44 [11.5]	4 [2.5]

Surgery was considered when was performed in the last 24 h before patients’ admission to the Intensive Care Unit. M- Male; F-Female; MV – Invasive mechanical ventilation; Cr Cl – Creatinine Clearance; Vasop – Vasopressors; Charlson – Charlson comorbidity score; SAPS II – Simplified acute physiology score II; SOFA – Sequential organ failure assessment score; IQR – Interquartile range; NA-Not available.

### Sampling

Sampling was performed within the first 36 hrs after starting antibiotic therapy (early samples) and repeated, whenever possible, in the 5^th^ or 6^th^ day of therapy (late samples). Five mL blood samples were collected into heparin-lithium test tubes immediately before the beginning of infusion and after 15, 30, 45, 60, 90, 120, 180, 360 and 480 min of the start of antibiotic infusion, which covers the times of expected peak and trough drug concentrations. The exact time of collection of the sample was registered. Blood samples were immediately centrifuged at 3000 rpm (roughly 1000*g) and at 4°C, during 10 min. Two mL of plasma aliquots were separated into polypropylene tubes containing an equal volume of 3-(N-morpholino) propanesulfonic acid (MOPS) as stabilizing solution. The mixture was immediately frozen at -40°C before being transferred into -80°C (within 48 hrs), where were kept pending analysis. Drug quantifications were made within 3 months as of collection.

### Analytical determinations

The concentration of meropenem in plasma was determined by high-performance liquid chromatography (HPLC). Separation was performed at 35°C using a XTerra^®^ MS C18 cartridge (Waters, inc.) equipped with a Symmetry^®^ C18 guard column (Waters, inc.). The UV detection was performed at 300 nm.

At the time of analysis, samples were thawed at room temperature. One mL of plasma sample spiked with ertapenem (as internal standard) was loaded into the cartridge. The cartridges were washed two times with 1 mL of phosphate buffer and eluted with 1 mL of acetonitrile. The eluted solutions were evaporated under vacuum, at room temperature. The residue was dissolved in 60 μL of pure water and injected into the HPLC system.

The mobile phase consisted of a mixture of 92% phosphate buffer (pH 7.4) and 8% acetonitrile pumped at 1 mL/min. The autosampler temperature was kept at 4°C and the injection volume was 5 to 25 μL.

This method showed to be linear over a range of 0.35-100 mg/L of meropenem concentration with a correlation coefficient always >0.998. Intra-assay accuracy ranged from -5.5% to -1.8% and precision was less than 3.9%. Inter-assay accuracy ranged from -8.1% to -1.4% and precision was less than 4.8%. The lower limit of quantification was 0.35 mg/L.

The method has also showed to be sensitive and specific in plasma samples obtained from intensive care patients not receiving meropenem, but a large number of other drugs commonly used in critically ill patients (including sedatives, vasopressors and other antibiotics).

### Pharmacokinetics

**Figure 1:**
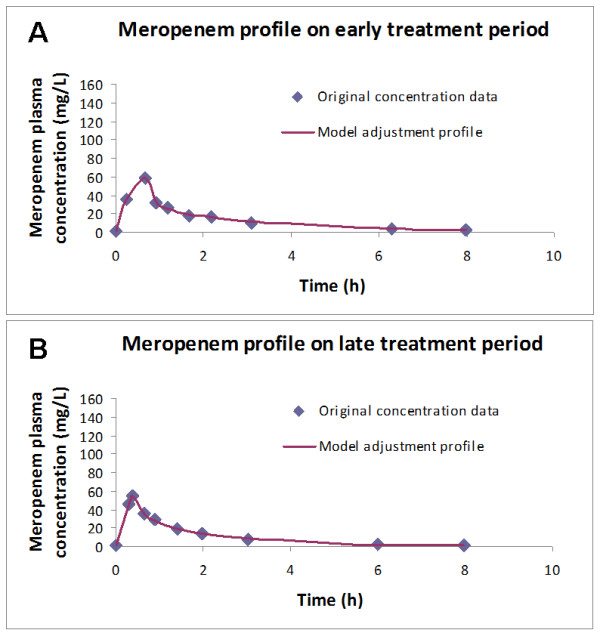
**Concordance between predicted and observed meropenem concentration profile in patient #11, either in the early (panel A) or in the late phase (panel B) of therapy.** The model was considered to be well adjusted.

Data were analyzed by WinNonlin 5.0.1 software (Pharsight Corp., Mountain View, California). A two-compartment model with zero order input and first order elimination was fitted into meropenem plasma profiles, using the least squares method. The model is considered to be well adjusted, with a mean r^2^ of 0.95 (ranging from 0.77 to 1.00) – Figure 1. The following PK parameters were calculated: elimination half-life (t_1/2_), volume of distribution at steady state associated to the central (*V*_ss1_) and to the peripheral (*V*_ss2_) compartments, area-under-the concentration-time curve (AUC) and total serum meropenem Cl.

We also measured trough concentrations to assess the possibility of drug accumulation.

The relationship between both meropenem Cl and Vd on one hand, and clinical relevant characteristics on the other hand, especially Cr Cl, SOFA score and Charlson comorbidity score, were assessed. A second set of late samples were collected whenever possible. We evaluated the differences between early and late meropenem PK to evaluate its relationship with patients’ improvement.

Finally we evaluated ability of conventional dose of meropenem to achieve a time over minimal inhibitory concentration (T > MIC) of 100%, assuming a MIC of 2 mg/L (the European Committee on Antimicrobial Susceptibility Testing (EUCAST) for *Pseudomonas aeruginosa*).

### Statistical analysis

Descriptive statistical analysis was performed. Continuous variables were expressed as median [interquartile range]. The PK parameters were expressed as geometric mean (95% confidence interval of the mean) to account for the log distribution of the data.

Correlations between severity and comorbidity scores with either *V*_ss_ or *V*_ss2_ and between Cr Cl and meropenem Cl were assessed using the Spearman rank correlation test and paired samples were assessed by the Wilcoxon signed rank test according to the data non normal distribution.

Data were analyzed using PASW Statistics v.18.0 (SPSS, Chicago, IL). All statistics were two-tailed, and significance level was defined as *p* <0.05.

## Results

### Early meropenem pharmacokinetics

Fifteen critically ill patients (eight male, median age 73 [21] yrs) were included in the study. Their clinical and demographic data are presented in Table 1.

Despite their old age, their clinical severity was relatively low with a median SOFA score of 4 and a SAPS II score of 44. Ten patients were receiving invasive mechanical ventilation and five were on vasopressors at the time of the first meropenem measurement. Seven patients were submitted to an abdominal surgery before enrolment. One patient died still in the ICU.

Individual and geometric mean of PK parameters measured at the first 36 hrs of antibiotic therapy are shown in Table 2. The geometric mean of *V*_ss_, normalized to patients’ weight, at the early stage of treatment was 0.20 (0.15-0.27) L/kg. No significant correlation of *V*_ss_/weight was found with either SOFA score (r^2^ = 0.25, p = 0.068), SAPS II (r^2^ < 0.01, p = 1.0) or Charlson comorbidity score (r^2^ = 0.06, p = 0.389). However the SOFA score was correlated with the *V*_ss2_/weight (r^2^ = 0.55, p = 0.002). Meropenem Cl geometric mean was 73.3 (45–120) mL/min, which was significantly correlated with the measured Cr Cl (r^2^ = 0.35, p = 0.033).

**Table 2:** Initial meropenem pharmacokinetic parameters

**Patient N°**	***V***_**ss**_	***V***_**ss**_**/weight**	***V***_**ss2**_**/*V***_**ss**_	**AUC**	**Cl**	**T**_**1/2**_	**Peak**	**Trough**	**T > 2 mg/L**	**T > 4 mg/L**	**T > 8 mg/L**
	L	L/Kg	%	L/mg.h	mL/min	h	mg/L	mg/L	100%	90%	70%
1	19.6	0.25	50.6%	157.1	106.7	2.9	63.3	3.0	100%	100%	100%
2	NA	NA	NA	264.7	63.3	1.5	NA	11.9	100%	100%	100%
3	13.6	0.21	56.3%	253.3	65	2.6	94.1	8.6	100%	100%	85%
4	13.6	0.17	78.4%	160.1	103.3	1.7	102.3	4.8	100%	85%	50%
5	13.4	0.16	51.8%	129.2	128.3	1.3	80.1	2.7	100%	90%	65%
6	32.7	0.42	93.7%	134.9	123.3	3.4	52.0	3.0	100%	100%	100%
7	18.6	0.23	73.6%	479.2	35	6.4	84.7	NA	100%	100%	100%
8	14.9	0.25	88.7%	465.9	35	5.0	91.3	NA	100%	100%	75%
9	6.7	0.07	90.4%	232.5	71.7	1.4	192.5	4.4	100%	75%	50%
10	13.0	0.16	58.4%	139.5	120	1.5	85.1	2.4	100%	75%	50%
11	20.9	0.30	68,6%	107.3	155	1.9	58.6	2.9	100%	100%	100%
12	13.6	0.18	79.2%	304.2	55	3.5	119.8	8.6	75%	50%	35%
13	17.1	0.27	34.9%	81.4	205	1.3	56.9	0.6	100%	100%	55%
14	22.5	0.28	53.1%	120.7	138.3	2.1	48.4	4.1	100%	100%	100%
15	13.0	0.13	68.6%	315.3	53.3	3.3	124.2	12.5	100%	90%	70%
**Geometric mean**	15.7	0.2	63.1%	190.2	73.3	2.3	85.9	3.8	100%	100%	100%
**95% CI**	12.7-19.4	0.15-0.27	52.7-75.5%	138.4-261.4	45-120	1.8-3.1	69.2-106.6	2.3-6.2			

*V*_ss_ – Volume of distribution at steady state; *V*_ss2_ - Volume of distribution at steady state (peripheral compartment); AUC – Area under the concentration-time curve; Cl – Meropenem clearance; T_1/2_ – Half life; NA- Data not available; IQR – Interquartile range; 95% CI - 95% confidence interval of the mean; T > - Percentage of time that meropenem concentration was above 2, 4 and 8 mgL.

Fourteen (93%) patients had meropenem concentrations at 8 hrs above the European Committee on Antimicrobial Susceptibility Testing (EUCAST) for *Pseudomonas aeruginosa* (2 mg/L) and therefore had a T > MIC of 100%. Only one had a trough concentration below that threshold (Table 2). Six patients had a trough concentration higher than 8 mg/L (4 times above the same threshold).

No adverse effects related to the antibiotic infusion were reported.

### Evolution of meropenem pharmacokinetics

In seven patients a late set of samples were collected. Early discharge from the ICU (3 patients), de-escalation of the antibiotic therapy (3 patients), incomplete data (1 patient) and withdrawal of consent (1 patient) precluded the completion of a second PK profile in the 8 patients.

In this subset of patients, the *V*_ss_/weight slightly decrease, from 0.25 (0.17-0.36) L/kg to 0.23 (0.1-0.53) L/kg from the early to the late set of measurements (Table 3). This difference was associated with a relative decrease of the weight of the *V*_ss2_, roughly 10% in 5 days, from 61.3 (42.5-88.5)% to 51.7 (36.6-73.1)%. Significant inter-patient variability was again noted.

**Table 3:** Comparison of early and late clinical and pharmacokinetic parameters in the 7 patients who completed two pharmacokinetic assessments

	**Early**	**Late**	P-value*
**Cr Cl** (mL/min)	66.7 [31.7]	106.7 [46.7]	0.128
**SOFA**	6 [3.5]	3 [1]	0.042
***V***_**ss**_ (L)	18.5 (13.0-26.4)	17.3 (7.3-41.0)	0.866
***V***_**ss**_**/Weight** (l/kg)	0.25 (0.17-0.36)	0.23 (0.1-0.53)	0.866
***V***_**ss2**_**/*V***_**ss**_ (%)	61.3 (42.5-88.5)	51.7 (36.6-73.1)	0.176
**Cl** (mL/min)	120 (75–188.3)	135 (73.3-228.3)	0.398
**Trough** (mg/L)	2.6 (1.1-6.4)	1.5 (0.4-5.9)	0.172

Cr Cl – Creatine Clearance; SOFA- Sequential organ failure assessment score; *V*_ss_ – Volume of distribuition at steady state; *V*_ss2_ – Volume of distribution at steady state of the peripheral compartment; Cl – Meropenem Clearance; Early – Parameters measured within the first 36 hrs of meropenem therapy; Late – Parameters measured after the 5^th^ day of meropenem therapy. *Wilcoxon signed ranks test. Data presented as geometric mean (95% confidence interval of the mean) or median [IQR].

In these 7 patients no accumulation of meropenem was found from the early to the late set of samples. However in 4 of them a trough concentration below 2 mg/dL was noted, probably related with the improvement in renal function. This translated in a lower T > MIC at this time point.

## Discussion

In this study we evaluated the PK of meropenem in 15 septic critically ill patients. We found important heterogeneity of both Cl, which parallels Cr Cl, and of *V*_ss_. Moreover, we noted a relative decrease of *V*_ss2_, in parallel with patients’ improvement (assessed by the SOFA score). Despite the variability of the PK parameters, only one of our 15 patients had a trough level lower than 1 mg/L in early samples and no significant meropenem accumulation between early and late samples were noted.

Similarly to our findings, the clinical studies addressing meropenem PK in septic patients have generally reported high *V*_ss_ and Cl, with large inter-patient variability, exceeding a twofold variation [10-12]. This variability of PK parameters in septic patients treated with meropenem was noted both in the same patient during infection treatment and between different patients [10].

The mean meropenem *V*_ss_ in patients with ventilator associated pneumonia has been reported to be as high as 0.47 L/kg [12] or as low as 0.11 L/kg [11]. In our study the geometric mean *V*_ss_ was 0.20 L/kg, which is in the range of the values reported in volunteers [1], although we noted large inter-patient variability (Table 2).

The β-lactam antibiotics are hydrophilic drugs usually eliminated by the kidney. In septic critically ill patients Cr Cl is commonly aumengted and this has been shown to occur in septic surgical or trauma patients [13] as well as in medical patients [14]. Moreover patients with normal plasma creatinine frequently have aumengted Cr Cl that may be unrecognized without direct measurement [15].

Meropenem Cl has been noted to be correlated, as in our study, with the Cr Cl [16] and increases in drug Cl may lead to underexposure and facilitate the emergence of resistance, especially when long antibiotic courses are used. Nevertheless the relationship with Cr Cl is not linear and changes in Cr Cl may not reliably predict variations in β-lactam PK [17]. In a study addressing 11 surgical patients no change in meropenem Cl was noted between the first and the fourth day of therapy, despite an increase of roughly 25% in Cr Cl [18], which was similar to our results. In the same cohort the authors again noted a decrease, although non significant, of meropenem mean *V*_ss,_ from 0.22 ± 0.06 to 0.17 ± 0.06 L/kg, accompanying clinical improvement [18]. In another study, addressing 25 critically ill patients (either from the ICU or from hemato-oncology) [19], the authors noted low trough concentrations and T > MIC due to increased Cl and Vd. Again these differences were only partly explained by increased Cr Cl. Conversely, drug accumulation occurred in ICU subjects with decreased renal function and therapeutic drug monitoring (TDM) was advised [19].

Since β-lactam TDM is not widely available, population PK models have been proposed. Moreover the model was improved in one study [20] by the inclusion of amikacin TDM and correctly predicted Vd and Cl of 4 different β-lactam antibiotics. In another study of population PK [21] imipenem Cl was found to be correlated with patients’ demographic characteristics (age, weight and height) as well as with Cr Cl.

In our study we were able to unveil a relative decrease of the *V*_ss2_ during treatment, which maybe consequence of the reversal of fluid shifts and decrease of the interstitial compartment fluid volume [22].

Changes in PK may lead either to sub-therapeutic concentrations or to drug accumulation. In a study, 25% of patients with severe sepsis or septic shock did not attained the intended target after the first dose of 1 g of meropenem; this was due to a large *V*_ss_ (0.43 [Interquartile range 0.43] L/kg) [23]. Also an increase in Cl and a lower T > MIC of β-lactam antibiotics, may follow the increase in Cr Cl, noted in several septic critically ill patient, and contributed to treatment failure [24]. Several of these studies excluded patients with the lower Cr Cl (either measured or estimated). On the contrary, we choose to include all critically ill septic patients in order to increase the external validity. However we aknowledge that this may also help to explain why we only found one patient with augmented renal [25] or meropenem Cl.

To overcome the altered PK of critically ill patients TDM has been proposed [5]. However, currently therapeutic target concentrations are poorly defined and β-lactam TDM is seldom available in most hospitals. Proposed targets of β-lactam antibiotics ranged between 40 to 60% of T > MIC [26] but a T > MIC as high as 100% [27] or 40% T > 4*MIC [23] has also been suggested. Acording to our findings, the use of TDM seems to be not usefull in an unselected population of critically ill septic patients. In fact, only one of our patients had a Cr Cl higher than normal, above 130 mL/min (Table 1). However that same patient was the only one who did not attained a T > MIC of 100%. Besides, we did not find evidence of either underdosing or drug accumulation between early and late measurements.

However we believe that this strategy may be usefull for selected patients at high risk of PK changes, particularly those with augmented renal clearance [28], although better definition of the target concentrations is probably needed.

Continuous infusion of β-lactam antibiotics has also been proposed to achieve an improved concentration profile and a T > MIC of 100% [29,30]. This strategy, despite its biological plausibility, has produced disapointing results so far. Two recent randomized prospective studies both unveiled a non-significant decrease in hospital mortality with continuous infusion of β-lactam antibiotics, despite higher microbiological response. The first one included 60 patients treated with piperacillin/tazobactam, meropenem or ticarcillin [31]. Hospital mortality was 10% in the continuous group versus 20% in the intermittent arm (p = 0.47). Another study included 240 patients treated with a high dose of meropenem (6 g/day), either by continuous or intermittent infusion [32]. Hospital mortality was 17.5% vs 23.3%, respectivelly (p = 0.34). Similarly, a meta-analysis of another 14 prospective studies [33] and a retrospective matched case–control study of piperacillin/tazobactam [34], again failed to show a survival benefit. It should be noted that, if changes in the Vd and high MIC are not considered, with continuous infusion concentration of the antibiotic might be always under the MIC.

Overall it seems that both these strategies, continuous infusion of β-lactam antibiotics and TDM, are probably helpful in the presence of bacteria with a high MIC or a high inoculum or in the presence of augmented renal clearance [35], especially in the early phases of therapy [36].

This study has some limitations namely it is single center and included a relativelly small number of patients. Beside we did not measured patients weight daily although we were not able to find a correlation between patients’ fluid balance and Vd. Nevertheless it also had some strengths: only critically ill septic patients were included, different infection focus were studied and an evaluation of early as well as late PK parameters was performed.

In the present study we confirmed the PK adequacy of the commonly used dose of meropenem to treat an unselected population of septic critically ill patients not receiving renal replacement therapy. As a result we did not find any evidence that the generalized use of meropenem TDM would be useful or cost-effective. Identification of sub-groups of patients most likely to benefit from this pratice should be performed before the general use of TDM monitoring can be recommended.

## Conclusions

In a population of septic critically ill patients meropenem PK was found to have important heterogeneity, especially Cl and *V*_ss_. A decrease of *V*_ss2_ was noted to parallel patients’ improvement in the second meropenem PK assessment. Trough levels were found to be above 2 mg/dL in almost all patients at early samples but only in half of patients in late samples.

## Abbreviations

AUC: Area under the concentration time curve; Cl: Clearance; Cr: Creatinine; ICU: Intensive Care Unit; HPLC: High performance liquid chromatography; MIC: Minimum inhibitory concentration; PK: Pharmacokinetic; SAPS: Simplified acute physiology score; SOFA: Sequential organ failure assessment; T>MIC: Antibiotic concentration time over bacteria MIC; t1/2: Half-life; TDM: Therapeutic drug monitoring; Vss: Volume of distribution at steady state; Vss1: Volume of distribution of the central compartment at steady state; Vss2: Volume of distribution of the peripheral compartment at steady state.

## Competing interest

J.G.P. has received honoraria and served as advisor of Pfizer, Astra-Zeneca, Gilead, Abbott, Wyeth-Lederle, Janssen-Cilag, Merck Sharp & Dohme P.P. has received honoraria and served as advisor of Astra Zeneca, Ely-Lilly, Gilead, Janssen-Cilag, Merck Sharp & Dohme, Novartis and Pfizer. All other authors had no competing interests to declare.

## Authors’ contributions

JGP conceived the study. JGP, NES and PP participated in the original design and in writing the original protocol. JGP and PP collected the samples and clinical data. NES, AM and CP developed the HPLC methodology, performed laboratory testing and pharmacokinetic modelling. All authors analysed data. JGP, NES and PP draft the manuscript. All authors have read and approved the final manuscript.

## Pre-publication history

The pre-publication history for this paper can be accessed here:

http://www.biomedcentral.com/2050-6511/15/21/prepub
